# A Nutritional Supplement (DìLsh^TM^) Improves the Inflammatory Cytokines Response, Oxidative Stress Markers and Clinical Signs in Dogs Naturally Infected by *Leishmania infantum*

**DOI:** 10.3390/ani10060938

**Published:** 2020-05-29

**Authors:** Vincenzo Mastellone, Nadia Musco, Giuseppe Vassalotti, Diego Piantedosi, Alessandro Vastolo, Monica Isabella Cutrignelli, Domenico Britti, Laura Cortese, Pietro Lombardi

**Affiliations:** 1 Department of Veterinary Medicine and Animal Production, University of Naples Federico II, 80137 Napoli, Italy; vincenzo.mastellone@unina.it (V.M.); nadia.musco@unina.it (N.M.); dapiante@unina.it (D.P.); alessandro.vastolo@unina.it (A.V.); monica.cutrignelli@unina.it (M.I.C.); pilombar@unina.it (P.L.); 2 Department of Health Sciences, University Magna Graecia, Catanzaro, 88100 Catanzaro, Italy; giuseppe.vassalotti@unicz.it (G.V.); britti@unicz.it (D.B.)

**Keywords:** leishmaniosis, dogs, nutraceuticals, oxidative stress, cytokines, leptin

## Abstract

**Simple Summary:**

The effects of a commercial nutraceutical supplementation in dogs naturally infected by *Leishmania infantum* were evaluated. Such a nutraceutical supplement was proposed to be added to the dog diet in order to improve the physiological immune response during chronic leishmaniosis. The present study suggests that such a supplement possesses anti-inflammatory and antioxidant properties that can lead to a clinical improvement in dogs naturally infected by *Leishmania infantum.*

**Abstract:**

The possibility to associate nutraceuticals, as immune-modulating tools, to the treatment of visceral leishmaniosis is a matter of great interest. In this study, we investigated whether the administration of a nutritional supplement (DìLsh^TM^, Dynamopet SRL, Verona, Italy) was able to exert beneficial effects on the inflammatory state and oxidative stress of the dogs naturally infected by *Leishmania infantum*. To this purpose, specific parameters, namely Tumor Necrosis Factor -alpha (TNFα), Interleukin-6 (IL-6), Inteleukin-10 (IL-10), leptin, derivates of Reactive Oxigen Metabolites (d-Roms) and Biological Antioxidant Potential (BAP), as well as the haematological and biochemical profiles of the infected dogs, were investigated upon the treatment with the nutritional supplement and compared with the conventional pharmacological anti-*Leishmania* therapy. The animals underwent complete clinical examination and blood sample collection before (T0) and 3 months after (T90) the onset of the two treatments. The two treatments showed similar results: significant clinical improvement, ELISA positivity and IgG decrease, an increase in IL-10, and a decrease in IL-6 were observed in animals treated with the nutritional supplement. A decrease in d-Roms and an increase in BAP were also detected in both groups. On the whole, the nutritional supplement possesses anti-inflammatory and antioxidant properties, suggesting that it may support animals’ health and be useful to extend the time a drug therapy is needed.

## 1. Introduction

Canine leishmaniosis (CL) is caused by protozoans of the species *Leishmania infantum,* which are transmitted by the bite of infected female phlebotomine sand flies. CL is one of the most important zoonotic disease affecting dogs and humans in many parts of the world (Mediterranean basin, South and Central America, and parts of Asia) [1]. Infected dogs can remain asymptomatic or progress towards an oligo-poly symptomatic disease, due to the pathogenic mechanisms of *Leishmania* and the variable immune response of individuals. 

In recent years, the use of natural remedies in alternative or in combination with the recommended drug therapy has been widely proposed in Western countries. Different herbal remedies are often used together in order to improve their beneficial effects. Indeed, some authors suggested that a synergic effect may occur using different substances both of natural and/or synthetic origin. Moreover, many attempts to identify the active components of herbal remedies have concluded that, in general, no one component is responsible for the therapeutic capacity, but rather a complex and intricate interaction of various herbs may result in therapeutic efficacy [2]. The use of plant-derived nutraceuticals may regulate immune response [3] and improve the clinical outcome of infectious diseases in both human and canine models [4,5,6,7]. Cortese et al. [8] reported that the combination of nutraceutical pet food with conventional therapy may modulate the immune response in canine leishmaniosis (CL). Two years later, Segarra et al. [9] reported the clinical efficacy of a treatment with dietary nucleotides and an active hexose correlated compound in addition to N-methylglucamine antimoniate in dogs with leishmaniosis. Sesquiterpene (-)-α-bisabolol has been described to be effective in regulating the Th1 response and in inducing clinical improvement in CL [10]. Moreover, in a recent study, Lombardi et al. [11] suggested that a nutraceutical supplementation was associated with immunomodulation of the Th1 response and a clinical improvement of the animals. Therefore, a potential supportive role of the nutraceutical supplement during canine leishmaniosis was proposed.

In the present study the efficacy of the administration of a commercial nutraceutical supplementation in dogs naturally affected by *L. infantum* was evaluated. This commercial supplement, DìLsh^TM^ (Dynamopet^TM^, Verona, Italy), is an association of Krill oil, 3%; dry mushrooms (*Cordyceps sinensis*), 2%; Krill flour, 1%; Gentian (*Gentiana Lutea* L.) dry root; and products obtained from the transformation of herbs (*Eleutherococcus senticosus* L.). The supplement, designed for dogs and cats, claims to support the natural physiological defences of the animal subjected to external aggressions. *Cordyceps sinensis* L. is recognized as an important medicinal mushroom in traditional Chinese medicine and is utilized for its properties. The traditional use of *Cordyceps sinensis* L. is to protect the kidneys [12], and it is well known that in the course of leishmaniasis, a loss of kidney function occurs. Indeed, recent researches have confirmed that *Cordyceps sinensis* L. possesses wide-ranging beneficial health effects, in particular a great anti-oxidation activity [13] that modulate the immune response [14], reduces the proliferation of cancer cells [15], improves hepatic function [16], reduces plasma cholesterol levels [17], and has hypotensive and vasorelaxant activities [18]. The Krill oil is obtained by an extraction process from *Euphausia superba* (Antarctic krill); it is rich in astaxanthin, which exerts an anti-oxidative effect, keeping intact the ω-3 polyunsaturated fatty acids (PUFA) and thus preserving them from oxidation [19]. Numerous studies, both in animals and in humans, demonstrated the health benefits of PUFA, in particular EPA (C20:5 ω-3) and DHA (C22:6 ω-3) [20], in terms of cardiovascular benefits and anti-inflammatory effects [21]. *Gentiana lutea* L., known as “great yellow gentian”, is an herbaceous perennial plant of the Gentianaceae family; it possesses digestive and purifying properties, particularly related to the root of the plant. The main active constituents of the plant are secoiridoids, iridoids, and xanthones, which exert the phytochemical properties. Recently, this plant has attracted much attention as a source of xanthone compounds that are known to exhibit a wide range of biological and pharmacological activities, e.g., antioxidative, hypoglycaemic, anti-viral, anti-bacterial, hepatoprotective activities [22]. *Eleutherococcus senticosus* L. belongs to the Araliaceae family, also known as Siberian or Russian ginseng, and is a medicinal herb with an impressive range of health benefits; it is primarily known as an adaptogen substance. This term describes a substance able to increase the resistance of the recipient to a variety of physical, chemical, or biological stressors [23].

In a previous study [11] on this nutraceutical, we showed an immunomodulating activity that did not provide an exhaustive explanation of the clinical improvement. For these reasons, in this study we explored the possible effects of DìLsh^TM^ on inflammatory and oxidative stress markers of 15 CL infected dogs, as compared with 10 CL dogs treated with anti-leishmanial therapy at diagnosis and during a three-month follow-up.

## 2. Materials and Methods

### 2.1. Ethical Statement

Dog owners provided formal consent to house the animals in adequate facilities, to treat their dogs with the nutritional supplement, and to take samples. All the procedures were approved and performed according to the Ethical Animal Care and Use Committee of the University of Naples Federico II (OPBA, CSV, protocol number 2017/0069148). This research avoided discomfort to the animals by the use of proper clinical management.

### 2.2. Nutraceuticals and Drugs

DìLsh^TM^ (Dynamopet SRL, Verona, Italy) is an association of Krill oil, 3%; dry mushrooms (*Cordyceps sinensis* L.), 2%; Krill flour, 1%; Gentian (*Gentiana Lutea* L.) dry root; and products obtained from the transformation of herbs (*Eleutherococcus senticosus* L.).

The reference control drug meglumine antimoniate (Glucantime^TM^, Merial) in combination with allopurinol was used.

### 2.3. Animals

Twenty-five privately owned dogs naturally infected by *L. infantum* were enrolled in the Campania and Calabria regions (South Italy), under the supervision of the veterinaries from the Department of Veterinary Medicine and Animal Productions of the University of Study Federico II (Naples, Italy) and from the Department of Health Sciences, University Magna Graecia (Catanzaro, Italy). Inclusion criteria were middle age (ranging from 5 to 7 years old) and a confirmed diagnosis of leishmaniosis based on (i) clinical and pathological signs of CL; (ii) LeiScan^TM^ enzyme-linked immunosorbent assay (ELISA) Test Rz > 1,1; and (iii) positivity of *L. infantum* DNA in sternal bone marrow (BM) aspirate, assessed by nested polymerase chain reaction (n-PCR). In turn, the following were excluded from the trial: pregnant or lactating dogs; dogs affected by severe hepatic or renal disease; dogs with concomitant infective diseases; and dogs under anti-*Leishmania* treatment in the last two years.

None of the animals received any drug therapy for at least four weeks before the onset and all along the experiment. At the recruitment (T0) and at the end of the trial (T90), the dogs were weighed and the Body Condition Score (BCS) was evaluated using a BCS scale of 1–9 points. Enrolled CL dogs were randomly divided into two groups (G, *n* = 10; and D, *n* = 15). Dogs from the G group were treated with meglumine antimoniate (50 mg/kg, subcutaneous, twice daily, for 1 month) and allopurinol (10 mg/kg, oral, twice daily, for 3 months). To the dogs of the D group, DìLsh^TM^ was orally administered in a ratio of 0.5 g/kg of live weight for 90 days. Dogs were evaluated before treatment (T0) and 3 months (T90) after the nutraceutical treatment. A complete clinical examination and the blood sampling were performed at T0 and T90. *Leishmania* DNA n-PCR detection was performed on sternal BM aspirates in enrolled dogs at T0.

To avoid any interference of environmental changing, the trial recruited only household dogs and the pet food were daily administered by the owners, as usual. DìLsh^TM^ supplementation was regularly added to the pet food by the same dog owner before the diet administration. All enrolled dogs carried out prophylaxis against flea, tick, and mosquito infestations through monthly local treatment with commercial products and were dewormed with a specific commercial product at the beginning of the trial.

### 2.4. Clinical Evaluation

Clinical evaluation of the enrolled dogs was performed by veterinarians according to the criteria adapted from da Silva et al. [24]:

Systemic signs—Attitude: active (0), apathetic (1); fever: absence (0), presence (1); lameness: absence (0), presence (1); nutritional status: normal (0), thin (1), cachectic (2); lymph nodes: normal (0), enlarged (1); mucosal colour: normal (0), pale (1); bleeding: absence (0), presence (1).

Cutaneous signs—Bristles: good (0), regular (1), bad/opaque (2); ear/nasal hyperkeratosis: absence (0), presence (1); nails: normal (0), long/onychogryphosis (1); skin lesion: absence (0), presence (1), ulcer (2); muzzle depigmentation: absence (0), presence (1); alopecia: absence (0), presence (1).

Ocular signs—Blepharitis: absence (0), presence (1); keratoconjunctivitis: absence (0), serous (1), mucopurulent (2).

All the individual scores were added to produce a total sign-based score ranging between 0 and 19 (15 signs). Occurrence of other illnesses or comorbidities was followed during the trial in order to exclude any positive dog. The clinical scores (CS) were obtained prior to treatments (T0) and at 3 months (T90).

### 2.5. Blood Sample Collection

Blood sample collection was cruelty free, without any blood operation and did not provide for any segregation of the animals. A total of 10 mL of blood was collected by jugular venipuncture after 12 h of fasting.

The total amount of blood was immediately divided into two fractions. The first fraction was placed in tubes containing potassium ethylene diamine tetra-acetic acid (EDTA) for a complete blood count (CBC), performed within 30 min from the collection. The other fraction was placed in plastic tubes, allowed to clot and centrifuged at 1200× *g* for 20 min in order to obtain blood serum. Serum samples were stored at −80 °C and defrosted immediately before the serological, biochemical, and oxidative stress and inflammation markers assays.

### 2.6. Diagnostic Procedure

The diagnosis of *Leishmania* was performed with the LeiScan^TM^ ELISA Test (Ecuphar, Barcelona, Spain) following the producer’s instructions and the optical density (OD) of each sample was measured at 450 nm using a microplate reader (Thermo Fisher Scientific, Waltham, MA, USA). Dogs were considered positive to be included in the study when the ratio (Rz) between the OD sample and the positive control OD was >1.1 [25]. *Ehrlichia canis* positivity was evaluated by IFAT using *E. canis* antigen in DH82 cells with a cut-off titre of 1:80. A sternal bone marrow aspirate for *Leishmania* and *E. canis* DNA detection by n-PCR was performed as described [26,27]. In G and D dogs, positivity for *Ehrlichia canis*, *Anaplasma phagocytophilum morulae*, and *Babesia canis* trophozoites and microfilariae in peripheral blood smears was excluded. Finally, *Dirofilaria immitis* infection was detected by the Snap Canine Combo Heartworm Antigen Antibody Test (IDEXX Westbrook, Maine, USA).

### 2.7. Complete Blood Count and Serum Biochemistry

A complete blood count was performed using a semi-automatic cell counter (Genius S, SEAC Radim Florence, Italy). Thrombocytopenia or evidence of platelet clumping was evaluated by May–Gruünwald–Giemsa-stained blood smears. Blood chemistry analyses were performed on serum aliquots by an automatic biochemical analyser (AMS Autolab, Diamond Diagnostics, USA) using reagents from Spinreact (Girona, Spain) to determine total proteins (TP), blood urea nitrogen (BUN), creatinine (CREA), alanine aminotransferase (ALT), gamma-glutamyl transferase (GGT), alkaline phosphatase (ALP), cholesterol (CHOL), and triglycerides (TRI). Finally, serum protein electrophoresis was also performed.

### 2.8. Measurements of Oxidative Stress and Inflammation Markers

Reactive Oxygen Metabolites-derived compounds (d-ROMS) and Biological Antioxidant Potential (BAP) were assayed by an automatic biochemical analyser (AMS Autolab, Diamond Diagnostics, Holliston, MA, USA) by using reagents from Diacron (Diacron International SRL, Grosseto, Italy). In the d-ROMs test, the reactive oxygen metabolites in the biological sample, in the presence of iron released from plasma proteins by an acidic buffer, are able to generate alkoxyl and peroxyl radicals according to the Fenton reaction. Such radicals can then oxidize an alkyl substituted aromatic amine (N, N-dietyl-paraphenylendiamine), thus producing a pink-coloured derivative that is photometrically quantified at 505 nm [28]. The d-ROMs concentration is directly proportional to the colour intensity and expressed as Carratelli Units (1 CARR U = 0.08 mg hydrogen peroxide/dL). In the BAP test, the addition of a plasma sample to a coloured solution, obtained by mixing ferric chloride solution with a thiocyanate derivative solution, causes a discoloration, whose intensity was measured photometrically at 505 nm and was proportionate to the ability of the plasma to reduce ferric ions [29]. The results are expressed as mole/L of reduced ferric ions. Both tests were validated for canine species [30].

Serum TNFα, IL-6, IL-10, and leptin were measured by canine ELISA kits (Genorise, Glen Mills, PA, USA). The TNFα detection range assay was 1–2200 pg/mL with the intra- and inter-assay CV < 7% and < 9%, respectively. The IL-6 detection range assay was 50–3200 pg/mL with the intra- and inter-assay CV < 6% and < 9%, respectively. The IL-10 detection range assay was 25–1600 pg/mL with the intra- and inter-assay CV < 5% and < 8%. The detection limit for leptin was 0.2 ng/L, and the intra-and inter-assay coefficients of variation (CV) were < 5%. Absorbance was determined using an automated microplate spectrophotometer (Epoch, BioTek Instruments Inc., Winooski, VT, USA) at 450 nm.

### 2.9. Statistical Analysis

Data were evaluated by Wilcoxon matched-pairs tests using GraphPad PRISM, version 8.0 (Graph-Pad Prism Inc, San Diego, CA, USA). Results were considered significant at *p* < 0.05. Results were expressed as the mean ± standard deviation (SD) unless indicated otherwise.

## 3. Results

**Figure 1:**
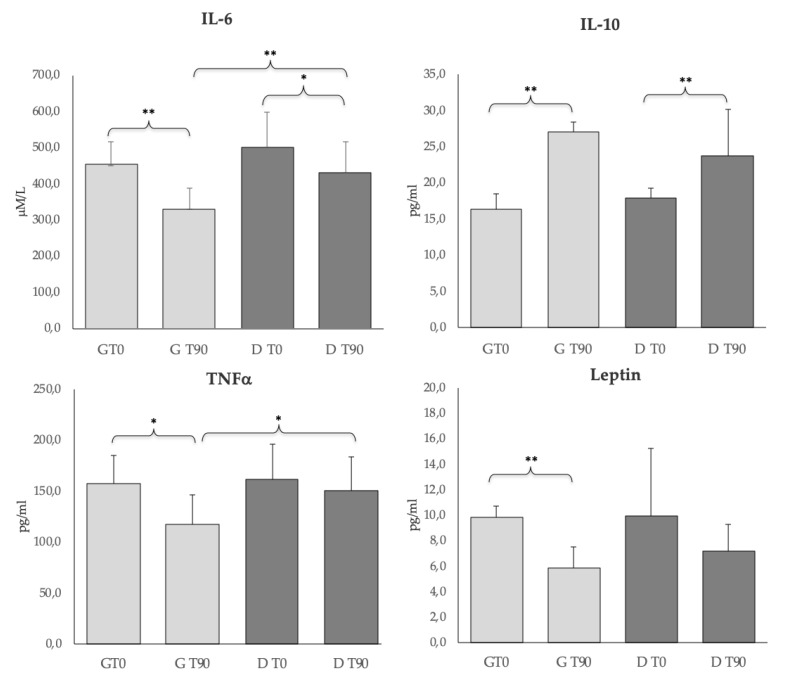
Tumor Necrosis Factor-alpha (TNFα), Interleukin-6 (IL-6), Inteleukin-10 (IL-10) and leptin levels in the G (*n* = 10) and D (*n* = 15) groups at T0 and T90. * *p* < 0.05, ** *p* < 0.01.

The BCS of both groups slightly improved after 90 days of treatments. In particular, at the time of recruitment (T0), the G group showed a mean body weight of 33.2 ± 15.6 kg and a mean BCS of 3.9, and at the end of the experimental period (T90) 36.5 ± 13.1 kg and a mean BCS of 4.2. The D group showed a mean body weight of 38.9 ± 18.6 kg at T0 with a mean BCS of 3.8 and at T90 43.2 ± 16.5 kg with a mean BCS of 4.3 on the BCS scale (1–9 points). Figure 1 shows the IL-6, IL-10, TNFα, and leptin levels in the G and D groups. Further, the D group presented a higher IL-6 than the G group (*p* < 0.01) and IL-10 significantly increased (*p* < 0.01) in both groups after the treatment. Further, a significant difference (*p* < 0.01) was seen for IL-6 at T90 between the groups. TNFα and leptin decreased only in group G after the treatment (*p* < 0.05 and *p* < 0.01, respectively). A significant difference (*p* < 0.05) was detected for TNFα between groups at T90.

**Figure 2:**
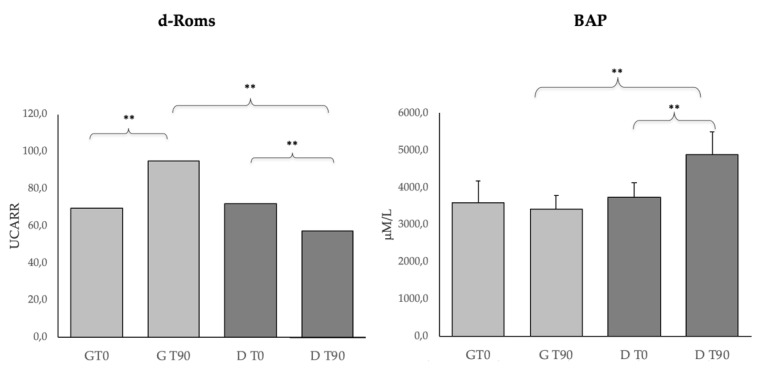
Derivates of Reactive Oxigen Metabolites (d-Roms) and Biological Antioxidant Potential (BAP) levels in the G (*n* = 10) and D (*n* = 15) groups at T0 and T90. * *p* < 0.05, ** *p* < 0.01.

Figure 2 shows the oxidative stress parameters (d-Roms, BAP) in the G and D groups. d-Roms significantly increased (*p* < 0.01) in the G group and significantly decreased (*p* < 0.01) in the D group after the treatment. Furthermore, a significant difference (*p* < 0.01) was seen between the groups at T90. The BAP levels did not change in the G groups while it significantly increased (*p* < 0.01) in the D group after the treatment. BAP was also significantly different (*p* < 0.01) between groups at T90.

The biochemistry parameters in the G and D groups are shown in Table 1. Significantly (*p* < 0.01) higher levels were seen for ALB in the G group after treatment, and between groups at T90. Decreased levels of g-globulins were detected in group G (*p* < 0.01) and D (*p* < 0.05) after the treatments; also, a significant difference was seen between groups at T90. No differences were detected for the other parameters. No differences were seen for the CBC parameters (data not shown).

**Figure 3:**
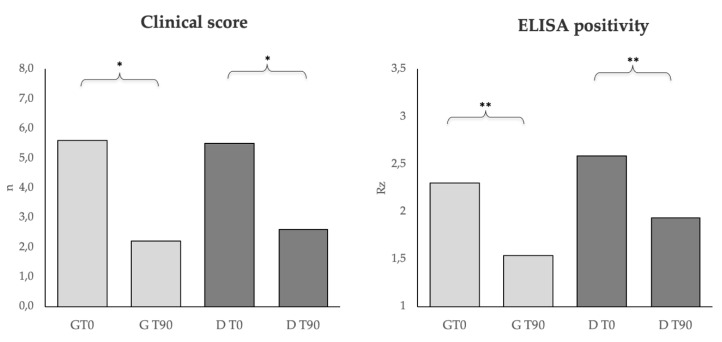
Clinical score and ELISA positivity in dogs belonging to the G (*n* = 10) and D (*n* = 15) groups at T0 and T90. * *p* < 0.05, ** *p* < 0.01.

Figure 3 shows the CS and the ELISA positivity in dogs belonging to the G and D groups at T0 and T90. The clinical scores, as well as the ELISA positivity, were improved (*p* < 0.01) in both groups after the treatment.

## 4. Discussion

Both treatments strongly improved the clinical scores and ELISA positivity (LeiScan^TM^); thus, DìLsh^TM^ seemed to achieve beneficial effects in terms of the dogs’ health, quite similar to those obtained using the pharmacological treatment. Regarding serum protein analysis by electrophoresis, an improvement was observed in both groups. Albumin, although it was in the reference range in all enrolled dogs, increased significantly at T90 (*p* < 0.01) only in the G group after pharmacological treatment, and between groups at T90. In addition, a significant decreased level of g-globulins was detected both in group G and D after treatments (pharmacological and nutraceutical), and a significant difference was seen between groups at T90. During CL, it is reported that the decrease in the levels of g-globulins starts to become evident not earlier than 4–6 weeks following treatment with antimonials [31,32]. The complete normalization of electrophoresis, however, could require at least 90–120 days [33,34]. In our study, this normalization was already evident at 90 days in both groups.

Clinical biochemistry, as well as haematology, showed that both treatments were well tolerated. The absence of adverse effects is often a critical point in the management of chronic diseases, both when using drugs or nutraceuticals. Indeed, since the adverse effects of nutraceuticals are often poorly explored, this result suggests that DìLsh^TM^ can be safely used in CL dogs.

The significant decrease in the IL-6 and the increase in the IL-10 observed in both groups after the two treatments confirm the efficacy of the conventional therapy and suggest the beneficial effects of the nutritional supplement. The cytokines are the most universal regulatory system in inflammation and host defence against bacterial infection [35,36]. They can participate in the immunopathologic process formation and function as diagnostic markers in some diseases. The significant reduction of TNFα seen only in the G group after treatment, the higher significance for IL-6 after treatment (*p* < 0.01 vs. *p* < 0.05, groups G and D, respectively), as well as the significant difference between groups for IL-6 at T90 show that the drug therapy was more active in modulating cytokines levels compared to the nutritional supplement.

Such an observation was also confirmed by the significant decrease of leptin in the G group after treatment. Leptin regulates the adaptive immunity, also influencing activities of T Helper (Th) 1 and 2 lymphocytes [37,38,39,40]. In particular, the hormone stimulates the Th1 production of proinflammatory cytokines, such as IL-2, interferon (IFN)-γ, TNFα, and IL-6, and inhibits a regulatory cell population (Treg), a CD3^+^CD4^+^CD25^+^ T lymphocyte subset, characterized by the expression of Foxp3 transcription factor [41,42,43]. In leishmaniosis, increased inflammatory response has been correlated with clinical exacerbation, and the immunotherapeutic role of Tregs appears to be relevant [44,45]. Tregs function, macrophage activation, and the proinflammatory state appear to be involved in the pathogenesis also of canine leishmaniosis. In naturally *L. infantum* infected dogs, an ineffective immune response to parasites appeared to be associated with a reduced percentage of Treg cells [46]. Di Loria et al. [47] showed an increase in leptin mRNA expression in dogs naturally infected by *L. infantum*. These immunological effects may be relevant in severe forms of CL in which alterations of the immune response (reduced circulating and increased tissue Tregs) [44,46] and subsequent persistent chronic inflammation could be the effect of a systemic increase in leptin expression. In our work, we reported a significant reduction in leptin levels only in group G (*p* < 0.01) after anti-*Leishmania* therapy. This positive reduction in Leptin levels could be related to the antiprotozoal activity of anti-*Leishmania* therapy.

Regarding the oxidative stress indices, the results of the d-ROMs and BAP tests suggest an antioxidant effect due to DìLsh^TM^ supplementation. This is important, since among the mechanisms that can influence health status, the balance of oxidative stress plays a relevant role [48]. At T0, the d-ROMs levels observed in both groups appeared within the “normal” range values (50 to 90 U CARR) proposed by Pasquini et al. [49] and by Sechi et al. [50]; whereas, the BAP levels appeared upwards from the “optimal” range values (2400–2200µmoli/L) proposed by the same authors. Interestingly, d-ROMs increased in the G group and decreased in the D group after the two treatments, whereas BAP increased only in the D group. These results testify a good antioxidant action of reactive species and could be related to the DìLsh^TM^ dietary factors that are involved in the prevention of the negative effects of oxidative stress [51]. The presence of antioxidants in DìLsh^TM^ might have opposed this oxidation stress, thereby preventing the oxidative damage they cause to the host’s biomolecules [48]. The significant differences observed between the groups at T90 could be related to the administration of a controlled and balanced antioxidant diet that may be a valid approach to restoring good cell metabolism and neutralizing excess free radicals in CL dogs [48].

The positive results obtained in our cohort of *L. infantum*-infected dogs after nutraceutical treatment are probably due to the healthy properties described for the components contained in the DìLsh^TM^ formula and, also, to a possible synergic effect between them, as suggested by Zhou et al. [52]. The similarity of the beneficial effects of both treatments in improving a leishmaniotic dog’s quality of life seems to be the results of the different mechanisms by which the two treatments act on their global health, impairing the protozoa responsible for the disease. By lowering the protozoa activity, the conventional therapy was much more effective than DìLsh^TM^ in reducing the negative effects due to the increase of some pro inflammatory molecules; on the other hand, together with the immune-modulating activity described by Lombardi et al. [11], the positive antioxidant effect of DìLsh^TM^ may exert important beneficial effects on a dog’s health status, finally resulting in similar clinical and clinicopathological results between the two treatments.

## 5. Conclusion

Overall, DìLsh^TM^ showed to be effective in CL dogs, improving inflammatory and oxidative markers and ameliorating clinical signs. Because of the increasing demand of consumers for natural products, also in veterinary medicine, it may be included in the therapeutic protocol to treat CL. Further studies may elucidate the mechanisms by which the different components of DìLsh^TM^ may act together to reach the best response. In CL, the possible use of DìLsh^TM^ to extend the time a new drug treatment is needed as well as the effects of a combined therapy with DìLsh^TM^ and conventional drugs should also merit further studies.

## Figures and Tables

**Table 1:** Serum biochemistry (mean ± standard deviation) in the G (*n* = 10) and D (*n* = 15) groups at T0 and T90.

Serum Biochemistry	Unit	G T0	G T90	D T0	D T90	GT0 vs. GT90	DT0 vs. DT90	GT0 vs. DT0	GT90 vs. DT90
BUN	mg/dL	45.7 ± 12.7	35.4 ± 8.9	42.3 ± 23.2	30.0 ± 11.1	NS	NS	NS	NS
CREA	mg/dL	1.14 ± 0.2	1.00 ± 0.2	1.04 ± 0.4	1.25 ± 0.9	NS	NS	NS	NS
ALT	U/L	33.7 ± 12.2	37.0 ± 8.9	34.1 ± 7.7	37.5 ± 10.1	NS	NS	NS	NS
ALP	U/L	99.7 ± 39.6	77.9 ± 24.5	77.2 ± 26.1	107.8 ± 79.8	NS	NS	NS	NS
GGT	U/L	3.1 ± 1.2	5.0 ± 2.2	4.3 ± 2.0	2.7 ± 1.5	NS	*	NS	*
TP	g/dL	7.2 ± 0.8	7.0 ± 0.6	7.7 ± 0.9	7.3 ± 0.7	NS	NS	NS	NS
ALB	g/dL	3.1 ± 0.4	3.8 ± 0.3	3.2 ± 0.5	3.4 ± 0.4	*	NS	NS	*
a1	g/dL	0.25 ± 0.06	0.20 ± 0.07	0.23 ± 0.08	0.19 ± 0.06	NS	NS	NS	NS
a2	g/dL	0.73 ± 0.17	0.88 ± 0.25	0.77 ± 0.26	0.95 ± 0.25	NS	NS	NS	NS
b1	g/dL	0.90 ± 0.15	0.87 ± 0.25	0.88 ± 0.43	0.94 ± 0.41	NS	NS	NS	NS
b2	g/dL	0.93 ± 0.23	0.77 ± 0.07	1.15 ± 0.34	1.01 ± 0.32	NS	NS	NS	NS
g	g/dL	1.31 ± 0.37	0.63 ± 0.42	1.47 ± 0.6	1.01 ± 0.34	**	*	NS	**
CHOL	mg/dL	172.3 ± 25.4	181.3 ± 10.5	192.7 ± 32.0	194.7 ± 48.3	NS	NS	NS	NS
TRI	mg/dL	68.6 ± 18.6	71.3 ± 17.3	84.1 ± 40.9	88.2 ± 61.1	NS	NS	NS	NS

BUN: blood urea nitrogen; CREA: creatinine; ALT: alanine aminotransferase; ALP: alkaline phosphatase; GGT: gamma-glutamyl transferase; TP: total proteins; ALB: albumins, a1,a2,b1,b2,g: globulins fractions; CHOL: cholesterol; TRI: triglycerides; NS = non-significant; * *p* < 0.05, ** *p* < 0.01.
